# Defining early health technology assessment: building consensus using Delphi technique: a commentary on implementation and diffusion of early HTA

**DOI:** 10.1017/S0266462325100214

**Published:** 2025-06-04

**Authors:** Linn Nathalie Støme

**Affiliations:** Department of Research and Innovation, Oslo University Hospital, Oslo, Norway

**Keywords:** Early HTA, health innovation, public-private collaborations, technology transfer, public procurement

## Abstract

This article presents the first consensus-based definition of early health technology assessment (HTA): “an HTA conducted to inform decisions about subsequent development, research, and/or investment by explicitly evaluating the potential value of a conceptual or actual health technology.” The definition was developed and refined through the involvement of relevant stakeholders in the field, a working group, and a survey panel, aiming to reach a consensus. An important part of this work was distinguishing between early HTA and related concepts, such as early awareness, dialogue, and scientific advice; thus, clarifying its unique role in HTA. Furthermore, the authors discuss how early HTA may guide investment decisions in development and reduce research waste. In addition, the consensus-based definition may enhance clarity for developers in producing early decision support to reach healthcare providers and policymakers. Finally, the article emphasizes the need for standardized terminology to increase the visibility of research, development, and policy in early HTA.

For the field of health technology assessment (HTA), the work presented by Grutters et al. and Bouttell et al. on the first consensus-based definition of early HTA is a significant advancement. The authors define early HTA as an assessment to inform decisions about subsequent development, research, and investment. This work promotes need-based development in health care before full-scale implementation. In Norway, early HTA has been a part of publicly funded research through multi-partner research centers for a decade. Norway has a publicly funded healthcare system with an increasing industry of private healthcare actors. Although the use of early HTA has been proven most beneficial in several research projects and scientific publications, the method is not integrated into the regulatory system for the evaluation and implementation of new health technology nationally. This proves the gap between evidence and practice, requiring efforts beyond bottom-up, case-based approaches. This consensus-based definition may offer a much-needed foundation for the implementation and diffusion of early HTA within existing processes in the healthcare system.

Moreover, uniformity of terminology may ease the integration of early HTA into widespread use in public–private collaborations, technology transfer, and public procurement (1–3).

## Early HTA as a tool in harmonizing needs in public–private collaborations

While many advancements in health innovation are nudged internally in the healthcare service, a non-negligible and crucial part consists of public–private collaborations. This form of joint effort bridges the gap between scientific research and commercial application (4). In this, we often see a distribution of roles where the public health service steers fundamental research with early-stage public funding. While the private health industry brings capabilities in product development, technological knowledge, and scaling strategies. In this crossroads of public and private interests, the integration of early HTA may help uncover and align the needs of the sectors to ease the implementation of emerging technology. A consensus definition is important in promoting the field of early HTA and establishing shared goals for evaluating the potential impact and feasibility of technology implementation.

A more formalized form of public collaborations is public–private partnerships (PPPs). This partnership model enables knowledge transfer, shared risk management, and streamlines the innovation processes, when applied properly (5). However, PPPs have evident challenges, including conflicting interests and transparency issues in data sharing. Early HTA can be used as an objective assessment tool to ensure that public funds are allocated to projects with the highest potential impact, thereby maximizing return on investment and mitigating risks for the involved parties (6). A more established use of early HTA to curate this form of joint development would benefit PPP models. For instance, public priority settings may be integrated into the early HTA process to ensure that private-sector developments align with broader health system goals, fostering socially sustainable innovation (7). In this, private interests such as public commitment to investment and market access could also be included in the development plan at an early stage. While the consensus-based definition of early HTA may not provide an ideal PPP model overnight, explicit terminology may greatly benefit the positioning of early HTA in private and public collaborations.

## Technology transfer and early HTA: bridging the gap between public and private sectors

An important product of PPPs is technology transfer, taking a technology from the research and testing stage in healthcare services, through commercialization, and finally reaching the patient’s bedside as a permanent part of care. This process enables collaboration between academic researchers, private industry, and public health institutions (8). This moving of technologies from research institutions into the healthcare system is an effective part of health innovation, which requires the alignment of several stakeholders (9). Universities, research institutions, and government agencies often develop innovative technologies, and private enterprises commercialize the technology to allow broader implementation. In this, a more widespread and structured use of early HTA may serve as a bridge between stakeholders by aligning development with healthcare priorities (3). Today, early HTA aids in this transition by offering a structured evaluation of market needs, cost-effectiveness, and integration challenges. For example, early HTA helps innovators refine product and service design and purpose in medical device development to meet user needs and identify viable funding sources to scale their solutions (2). Early HTA ensures that technology transfer decisions are based on robust evidence, maximizing the social impact of innovations (6). If the consensus-based definition can increase the integration of early HTA and spark future research and development, as the authors hope, this could directly benefit the implementation and commercialization of health innovations.

Furthermore, the regulatory process of technology transfer to ensure safety, efficacy, and ethical standards may be challenging and a barrier to innovation (10). In Norway, a workshop on the evaluation of digital innovation in healthcare revealed that this is the case for industry members in the technology cluster Norway Health Tech (11). Integrating more uniform structures in early HTA may help streamline the efforts and requirements in the regulatory process; thus, revealing potential hurdles early in the development and providing guidance on meeting regulations. In addition, by integrating value-based assessments, early HTA can ensure that technology transfer efforts prioritize patient-centered outcomes and long-term health system sustainability (12).

## Enhancing value-based procurement in public health care

The authors discuss how early HTAs help guide investment decisions and provide decision support to technology developers, healthcare providers, and policymakers. Integrating early HTA into public procurement may translate early findings into actionable healthcare decisions. By incorporating early HTA into procurement policies, governments, and health authorities can ensure that new technologies meet predefined needs before investment (13). Public procurements involve various stakeholders with different incentives. These include public policymakers, healthcare providers, patients, and industry. Early HTA may provide evidence-based criteria for evaluating needs, prioritizing solutions, and patient outcomes for technologies still under development. Thus, avoiding investments in faulty solutions increases long-term system sustainability. For instance, in value-based procurement models, priorities are not only based on the initial purchase price of a technology but also consider its broader impact on health system efficiency, service quality, and satisfaction (14).

A key advantage of embedding early HTA into public procurement is the potential to foster value-based decisions under budget constraints. Increased use of the discipline may allow us to identify promising innovations early in the development pipeline and provide guidance on their optimal implementation (1). The consensus-based definition may speed up the integration of early HTAs to support a more strategic approach to procurement in the public sector. On the industry side, stakeholders may benefit from more explicit market signals on costs and expected performance of the technology. This may reduce uncertainty and incentivize need-based development. Although uniform terminology in early HTA is not the only effort needed to achieve this, successful integration of early HTA into procurement frameworks will require institutional capacity building and policy alignment. However, the consensus-based definition represents an important stepping stone in this work. In addition, standardized assessment criteria and international collaborations are needed to enhance the consistency and impact of early HTA-driven procurement strategies (15).

## Conclusions

Early HTA is a promising tool to promote evidence-based development in health care. Its integration, from research to practice, in the healthcare system may depend on both knowledge transfer and incentive schemes. When it comes to the implementation and diffusion of early HTA, establishing a consensus-based definition of early HTA marks a critical step in advancing the field. Knowledge transfer of the method application and utility may increase as a result of the work on the consensus-based definition. This work’s enhanced clarity may ease strategic integration into public–private collaborations, technology transfer, and public procurement policies. By fostering collaboration between the public and private sectors, early HTA may be embedded into procurement decision-making, optimizing resource allocations and patient outcomes. This may lead to eligibility into proper incentive schemes in the long term. As the field of early HTA continues to evolve, ongoing research and policy developments will be essential to maximize its impact and ensure its role in driving sustainable and equitable healthcare innovation.
